# RCAN1 in the inverse association between Alzheimer’s disease and cancer

**DOI:** 10.18632/oncotarget.23094

**Published:** 2017-12-11

**Authors:** Qiang Fu, Yili Wu

**Affiliations:** ^1^ Department of General Surgery, Tianjin Medical University General Hospital, Tianjin, China; ^2^ Department of Psychiatry, Jining Medical University, Jining, Shandong, China; ^3^ Shandong Key Laboratory of Behavioral Medicine, Jining, Shandong, China; ^4^ Collaborative Innovation Center for Birth Defect Research and Transformation of Shandong Province, Jining, Shandong, China.

**Keywords:** RCAN1, Alzheimer’s disease, cancer, inverse association, Gerotarget

## Abstract

The inverse association between Alzheimer’s disease (AD) and cancer has been reported in several population-based studies although both of them are age-related disorders. However, molecular mechanisms of the inverse association remain elusive. Increased expression of regulator of calcineurin 1 (RCAN1) promotes the pathogenesis of AD, while it suppresses cancer growth and progression in many types of cancer. Moreover, aberrant RCAN1 expression is detected in both AD and various types of cancer. It suggests that RCAN1 may play a key role in the inverse association between AD and cancer. In this article, we aim to review the role of RCAN1 in the inverse association and discuss underlying mechanisms, providing an insight into developing a novel approach to treat AD and cancer.

## INTRODUCTION

Alzheimer’s disease (AD) is the most common type of neurodegenerative disease leading to dementia and aging is the major risk factor of AD development [1-2]. Cancer is one of the leading causes of morbidity and mortality worldwide and aging is also a fundamental factor in cancer development [3]. The inverse association between AD and cancer has been reported in several population-based studies [4-15]. Elucidating underlying mechanisms of the inverse association between AD and cancer will be beneficial to developing effective therapy for AD and cancer, particularly for AD as there is no effective treatment for AD. Recent studies indicate that regulator of calcineurin 1 (RCAN1) may play a key role in the inverse association between AD and cancer. For example, RCAN1 elevation promotes AD pathogenesis, while increased RCAN1 suppresses tumor growth [16-18]. Thus, we aim to review the role of RCAN1 in the inverse association and discuss underlying mechanisms, providing potential strategies for the treatment and prevention of AD and cancer by modulating RCAN1.

## RCAN1 GENE AND PROTEINS

Historically, RCAN1 has been named as DSCR1, MCIP1, ADAPT78, CSP1, DSC1 and RCN1. According to its conserved function of regulating calcineurin activity and calcineurin-dependent phenotypes in various types of cells and species, it was officially renamed as regulator of calcineurin 1 in 2007 [19-44] (Table 1). The *RCAN1* gene is located on chromosome 21, consisting of seven exons and six introns. RCAN1.1 and RCAN1.4 are the two major transcripts generated by alternative mRNA splicing, composing of exon 1, 5, 6, 7 and exon 4, 5, 6, 7, respectively[45]. RCAN1.1 is highly expressed in the brain, heart and skeletal muscle, whereas RCAN1.4 is predominantly expressed in the heart and skeletal muscle. The two transcripts are mainly translated into RCAN1.1L and RCAN1.4 isoforms with 252 and 197 amino acids, respectively. In addition, the transcript RCAN1.1 can also be translated into RCAN1.1S from the downstream translational start site by leaky scanning and reinitiation mechanisms although its expression is extremely low [18]. RCAN1.1and RCAN1.4 transcripts are differentially regulated at transcriptional level as their transcription is driven by differential promoters [45-46].

**Table 1:** General functions of RCAN1

Experimental condition	CaN interaction/ activity	Cells/species	Affected phenotypes	References
*In vitro* & *In vivo*	+	U2OS, COS-7, CHO, PC12, HEK293 Rat myocyte, mouse		[33, 37-41]
*In vitro*		HA-1	Cell proliferation	[42]
*In vivo*	+	S. cerevisiae		[121]
*In vitro &* *In vivo*	+	C2C12 Mouse myocyte	Cardiac hypertrophy	[43-44]
*In vitro*	+	C2C12		[133]
*In vitro*	+	HA-1 PC12	Cell death Cell proliferation	[130]
*In vivo*	+	Drosophila	Learning deficits	[26]
*In vitro &* *In vivo*	+	COS-7, CHO SH-SY5Y Mouse brain		[134]
*In vivo*	+	C. elegans	Calcineurin-deficient phenotypes: growth inhibition, small body size	[24]
*In vitro &* *In vivo*	+	BHK Mouse	Cell death	[135-136]
*In vivo*	+	Mouse	Cardiac hypertrophy	[137]
*In vivo*	+	Yeast		[21]
*In vitro*	+	E6-1	Cytokine expression	[138]

The interaction of RCAN1 with calcineurin (CaN) and the role of RCAN1 in regulating calcineurin activity/calcineuin-dependent functions are well conserved across species and cell lines, which has been well studied in multiple cell lines (e.g., COS-7, CHO, U2OS, HA-1, HEK293, SH-SY5Y, C2C12, BHK, primary neurons, HUVEC) and organisms (e. g., yeast, C. elegans, Drosophila, mouse) [20-44] (Table 1). As the expression of RCAN1 is extremely low in astrocytes and microglial cells, few study has been performed in glial cells [47-48]. However, it has to be noted that RCAN1 has dual role in regulating calcinurin activity depending on its level and phosphorylation status [33-34, 49]. For example, increased RCAN1 expression inhibits calcineurin activity, while low level of RCAN1 stimulates calcineurin activity *in vitro* [21, 49]. Calcineurin is a calcium/calmodulin dependent serine/threonine phosphatase, consisting of a catalytic subunit, calcineurin A, and a regulatory subunit, calcineurin B. By dephosphorylating NFAT, calcineurin promotes NFAT translocation into the nucleus contributing to a number of genes’ transcription and subsequent events, e.g., cell proliferation, apoptosis, angiogenesis, synaptic plasticity, immune response and skeletal/cardiac muscle development. Dysregulation or dysfunction of calcineurin has been linked to both AD and cancer, suggesting that RCAN1 may be involved in the pathogenesis of both AD and cancer via calcineurin-dependent pathways. On the other hand, RCAN1 interacts with multiple protein partners, such as integrinα_v_β_3_, NF-κB, ubiquitously-expressed prefoldin-like chaperone (UXT) and signal transducer and activator of transcription 2 (STAT2), which may contribute to the calcineurin-independent functions, including cell proliferation, apoptosis and angiogenesis [50-53]. Accumulated evidence indicates that RCAN1 may play an important role in the inverse association between AD and some types of cancer via both common and differential processes.

## ALZHEIMER’S DISEASE AND CANCER

### Alzheimer’s disease

AD is the most common cause of dementia, accounting for 50-75% of dementia [54]. Less than 5% cases are early-onset AD (EOAD), who develop AD before age 65. For example, patients with Down syndrome (DS), caused by trisomy of chromosome 21, inevitably develop of AD pathology after middle age. The majority AD cases are late-onset AD (LOAD), who develop AD after age 65 [2]. In 2010, World Alzheimer International estimated approximately 36 million people suffering from dementia worldwide and it costs US $604 billion. Due to the rapid increase in aging population, the AD prevalence is continuously increased worldwide and the costs will reach to US $ 1 trillion by 2030 [54]. Progressive memory loss is the characteristic of AD, while cognitive deficits and psychosis may also be presented [55-57]. Extraneuronal neuritic plaques, intraneuronal neurofibrillary tangles, and synaptic/neuronal loss leading to brain atrophy are the pathological characteristics of AD, while neuritic plaque is the unique feature of AD neuropathology [58-67]. Amyloid β (Aβ) and phosphorylated Tau are the major components of neuritic plaques and neurofibrillary tangles, respectively [66, 68-72].

### Cancer

Cancer is a group of diseases characterized with impaired cell growth control, poor differentiation and the potential to invade or spread to the other parts of the body although different types of cancer may be mediated by differential signaling pathways. It is one of the leading causes of death globally. Approximately 14 million new cases were diagnosed in 2012 and the number is expected to rise 70% over the next 2 decades [73]. 8.8 million people died from cancer in 2015 and the costs are US $ 1.16 trillion in 2010 [73]. Although aging is also a fundamental factor for cancer development, the inverse association between AD and cancer has been reported in a number of studies [4-7]. A significant improvement has been made in cancer prevention and treatment in the past decades. However, there is no effective treatment for AD. Thus, elucidating mechanisms of the inverse association between AD and cancer will be beneficial to developing effective therapy for AD and cancer, particularly for AD, as there is no effective treatment for AD.

### The inverse association between AD and cancer

The inverse association between AD and cancer has been reported in several population-based studies and meta-analysis [4-15]. For example, cancer history is associated with the delay and reduced risk of AD onset [14-15]. Musicco et al. reported that the risk of AD among patients with cancer was 35% reduced and the risk of cancer in patients with AD nearly halved [6]. In addition, the risk of occurrence was significantly reduced for lung and colorectal cancer although the five most frequent sites of cancer in patients with AD dementia was lower [6]. Realmuto et al. showed that frequency of cancers at sites of breast, uterus, ovary and skin was reduced in AD cases compared with that in the controls [9]. Freedman et al. reported that six cancer sites (e.g., rectum, breast, uterus, ovary, prostate, leukemia) were significantly and inversely related to AD [12]. However, the association between AD and site-specific cancers remains inconclusive according to the most recent meta-analysis and systematic review [7, 10, 74].

## DYSREGULATION OF RCAN1 IN ALZHEIMER’S DISEASE AND VARIOUS TYPES OF CANCER

Increased RCAN1 expression is detected in AD brains [17-18, 48]. Moreover, multiple risk factors may be involved in its upregulation. For example, glucocorticoid, increased in AD patients, upregulates RCAN1.1 transcription in addition to promoting Aβ generation and tau hyperphosphorylation [17, 75-82]. Apolipoprotine E (*ApoE4*) allele, a well-known risk factor of AD, significantly increases RCAN1 expression [83]. Ischemic stroke, a risk factor of AD, markedly increases RCAN1.4 expression [84-85]. In addition, *RCAN1* gene polymorphisms within the promoter region are associated with AD [86]. Moreover, NF-κB, an inflammatory molecule, could activate RCAN1 transcription and block its degradation, leading to its upregulation [87-88].

RCAN1 dysregulation has also been detected in some types of cancer and cancer cells. For example, RCAN1.4 expression is reduced in some cancer cell lines at both transcriptional and post-translational levels, e.g., melanoma and thyroid cancer cells [89]. RCAN1 expression is significantly lower in lymph node metastasis compared with that in the primary tumor in papillary thyroid cancer [90]. Recently, Jin et al. reported that RCAN1.4 expression is significantly reduced in hepatocellular carcinoma compared with that in the adjacent non-cancer tissues [91]. Metastin, a tumor metastasis suppressor, increases RCAN1.4 expression in thyroid cancer cells [90]. In addition, NFAT family members, downstream targets of RCAN1, are constitutively activated in several types of cancer, including breast cancer, pancreatic cancer, aggressive T cell lymphoma, Burkitt’s lymphoma, and diffuse large B cell lymphoma, suggesting that the inhibitory effect of RCAN1 on calcineurin/NFAT may be reduced by its downregulation [92]. However, increased expression of RCAN1.4 has also been detected in other types of cancer, such as hypopharyngeal cancer and Kaposi’s Sarcoma (KS) [93-94].

## MECHANISMS OF RCAN1 IN THE INVERSE ASSOCIATION BETWEEN ALZHEIMER’S DISEASE AND CANCER

In addition to facilitating Aβ generation and Tau phosphorylation in AD, growing evidence suggests that RCAN1 is involved in several common processes in AD and cancer, such as apoptosis, cell proliferation and angiogenesis [95-97] (Table 2). DS patients inevitably develop AD pathology after middle age and the incidence of cancer is different from the controls, including lower incidence of some types of solid cancer and higher incidence of leukemia [98-102]. However, DS is not a proper model to represent the role of RCAN1 in the association between AD and cancer because of the following reasons. Although the expression of RCAN1 is increased in DS patients by an extra copy of the *RCAN1* gene, it has to be noted that DS is caused by an extra copy of chromosome 21, which consists of more than 160 coding genes and a number of microRNAs in addition to the *RCAN1* gene. Thus, DS could not fully reflect RCAN1’s function. Moreover, many genes on chromosome 21 are implicated in cancer development, such as amyloid-β precursor protein (*APP*), superoxide dismutase 1(*SOD1*), dual specificity tyrosine phosphorylation regulated kinase 1A (*DYRK1A*) etc., suggesting that the alteration of the incidence of cancer in DS is attributed to the combined effect of multiple genes [103-105]. Therefore, the altered incidence of cancer in DS is not discussed in the manuscript.

**Table 2:** Mechanisms of RCAN1 in AD and cancer

Experimental condition		CaN interaction/ activity	Cells/species	Affected phenotypes	References
Apoptosis					
*In vitro*			Mouse primary neuron	Apoptosis	[139]
*In vitro*	+		Mouse primary neuron, SH-SY5Y	Apoptosis	[17-18, 107, 109]
*In vivo*			ST14A(neuronal)	Apoptosis	[106]
*In vitro*					
*In vitro*			Drosophila (neuron)	Apoptosis	[29]
			U87MG cells (human glioblastoma cells)	Apoptosis	[140]
*In vitro*			U251, T98G (glioma cells)	Apoptosis	[110]
*In vitro*			CEM, Nalm-6 (leukemia cells)	Apoptosis	[111-113]
*In vitro &* *in vivo*	+		Burkitt’s lymphoma	Apoptosis	[53]
Angiogenesis					
*In vitro &* *in vivo*	+		HUVEC Primary endothelial cell mouse	Angiogenesis: proliferation and tube formation	[16, 117-118]
*In vitro &* *in vivo*	+		HUVEC X. laevis	Angiogenesis: vascular branching	(Fujiwara et al., 2011)
Proliferation and migration					
*In vivo*			RCAN1 transgenic mice	Neurogenesis: Proliferation, migration, Maturation	[126]
*In vitro*			PC-12 cells (pheochromocytoma cells)	Proliferation	[130]
*In vitro*	+		MHCC97H, HCCLM3 (hepatocellular carcinoma cells)	Proliferation, Migration	[91]
*In vitro*	+		ARO, NPA (human thyroid carcinoma cells)	Proliferation	[90]
*In vitro*			ARO, WRO, NPA, FTC133	Migration	[89].
*In vitro*			U87MG (glioblastoma)	Proliferation	[129]
*In vitro &in vivo*			8505c, BCPAP, C643, FTC236 and SW1736 (Human thyroid cancer cell lines), mouse	Proliferation	[127]
*In vitro*			Ishikawa cells (endometrial adenocarcinoma)	proliferation	[128]

## RCAN1 PROMOTES APOPTOSIS

### RCAN1 promotes neuronal apoptosis in AD

Increased RCAN1-induced apoptosis promotes AD pathogenesis but suppresses the development and progression of cancer. Many studies indicate that increased RCAN1 expression plays a pivotal role in the pathogenesis of AD by promoting neuronal apoptosis. First, RCAN1.1S overexpression and chronic RCAN1.1L overexpression inhibit calcineurin activity and promote caspase-3-mediated neuronal apoptosis, while acute RCAN1.1L overexpression protects neurons from stress-induced apoptosis by inhibiting caspase-3 activity [17-18]. Chronic overexpression of RCAN1.1L and RCAN1.1S impairs the function of mitochondria by promoting its degradation and accelerating ATP-ADP exchange rate, respectively, contributing to neuronal apoptosis [106]. Increased RCAN1.1 promotes Aβ-induced neuronal apoptosis in Drosophila, while RCAN1.4 expression promotes calcium overloading-induced neuronal apoptosis *in vitro* [29, 107]. In addition, RCAN1 overexpression dramatically increases Tau phosphorylation and Aβ generation, which also contributes to neuronal apoptosis in AD [97, 108]. Moreover, increased RCAN1.1L is a key mediator in amyloid precursor protein (APP) overexpression-induced neuronal apoptosis, while APP elevation is involved in the pathogenesis of both familial AD and sporadic AD [109].

### RCAN1 promotes cell apoptosis in various types of cancer

Increased RCAN1 facilitates cancer cell apoptosis, which is a possible mechanism of inhibiting cancer development and progression. For example, increased RCAN1.1 or RCAN1.4 promotes lymphoma glioma cell apoptosis *in vitro* and *in vivo* by inhibiting the nuclear translocation of NF-κB [53, 110]. Moreover, RCAN1.1 is an important mediator in glucocorticoid-induced apoptosis in leukemia cells by downregulating and upregulating anti-apoptotic and pro-apoptotic proteins, respectively [111-113].

### RCAN1 suppresses angiogenesis

Alteration of angiogenesis is implicated in the pathogenesis of AD and cancer. Angiogenesis deficits involved in AD pathogenesis[114]. For example, vascular endothelial growth factor (VEGF), a factor facilitating angiogenesis, associates with optimal brain aging and might be a potential therapy against AD [115-116]. However, angiogenesis is a major mechanism of cancer development and progression. Accumulated evidence indicates that RCAN1 participates in endothelial cell migration and angiogenesis mediated by both calcineurin/NFAT dependent and independent signaling. Most studies indicated that RCAN1.4 inhibits angiogenesis *in vitro* or *in vivo*. For example, Minami et al. reported that constitutive expression of RCAN1.4 impairs endothelia cell proliferation and tube formation, leading to the inhibition of angiogenesis and tumor growth in mice [117]. Consistently, Yao et al. reported that RCAN1.4 could act as an inhibitor of angiogenesis by regulating calcineurin/NFAT signaling [118]. More importantly, Baek et al. found that mild increase of RCAN1 expression by an extra copy of *RCAN1* gene suppresses tumor growth by inhibiting tumor angiogenesis in mice [16]. In addition, RCAN1 inhibits vascular branching during angiogenesis *in vivo* [119]. The aforementioned evidence indicates that mild increase of RCAN1 expression may contribute to the inverse association between AD and some type of cancer by suppressing angiogenesis.

Several reports showed that increased expression of RCAN1.4 is associated with angiogenesis in hypopharyngeal cancer and Kaposi’s Sarcoma (KS) caused by Kaposi’s Sarcoma Herpesvirus (KSHV) [93-94], suggesting that increased RCAN1 expression may only be responsible for the inverse association of AD and some types but not all types of cancer. In addition, Ryeom et al. reported that RCAN1 knock-out inhibits angiogenesis and tumor growth in mice [120]. However, it has to be noted that low level of RCAN1 is necessary for calcineurin/NFAT activity [21, 49, 121].

### RCAN1 inhibits cell proliferation and migration

RCAN1 plays a pivotal role in cell proliferation and migration, which are implicated in both neurogenesis in AD, and tumor growth and metastasis in cancer [122-125].

#### RCAN1 inhibits neurogenesis in AD

Casas et al. first reported that RCAN1 is involved in neurogenesis, including neural progenitor cell proliferation, migration and maturation [123]. Moreover, the number of neurons within hippocampus is reduced in RCAN1 transgenic mice, which is associated with the defect in neural progenitor cell proliferation [126]. In addition, RCAN1 significantly inhibits neuronal maturation, which is characterized with reduced dendritic spines [126]. Thus, increased RCAN1 contribute to neurogenesis defect in AD by impairing neural progenitor cell proliferation, migration and maturation.

#### RCAN1 inhibits cell proliferation and migration in various types of cancer

Increased RCAN1.4 expression leads to growth arrest of fibroblast cells [42]. RCAN1.4 knockdown promotes tumor growths, which is mediated by nuclear factor erythroid 2-like 3 (NFE2L3) [127]. In addition, RCAN1-4 inhibits epithelial cell proliferation in endometrial adenocarcinoma via a negative regulation of C-X-C motif chemokine ligand 8 (CXCL8) [128]. Moreover, RCAN1.4, downregulated in hepatocellular carcinoma, prevents cancer cell proliferation and migration [91]. However, reduced RCAN1.4 expression attenuates proliferation of glioblastoma cells mediated by inhibiting Ras signaling, which is independent of calcineurin [129]. RCAN1.1S stimulates the proliferation of pheochromocytoma PC-12 cells [130]. The aforementioned evidence indicates that RCAN1 differentially regulates cell proliferation in various types of cancer. Moreover, RCAN1.4 could block cell migration to inhibit metastasis in papillary thyroid cancer [90]. Consistently, increased RCAN1 expression inhibits cancer cell migration, while loss of endogenous RCAN1 leads to an increase in migration in a couple of cancer cell lines, such as ARO, WRO, NPA and FTC133 [89]. Reduced RCAN1.4 expression are associated with advanced tumor stages and poor differentiation of hepatocellular carcinoma, while increased RCAN1.4 markedly reduces cancer cell proliferation and migration in hepatocellular carcinoma cells [91]. It suggests that RCAN1 could inhibit cancer development and progression by inhibiting both cancer cell proliferation and migration.

## THERAPEUTIC POTENTIAL OF REGULATING RCAN1 FOR THE TREATMENT OF AD AND CANCER

Although accumulated evidence indicates that RCAN1 might be a potential target for the treatment of AD and certain types of cancer, currently, no drug is developed based on the regulation of RCAN1. To develop drugs based on RCAN1 regulation, the following issues should be considered. First, calcineurin inhibitors have been widely used as the immunosuppressive drug in the organ transplantation and certain types of autoimmune disorders. Thus, the role of RCAN1 in immune function should be considered, which is implicated in both AD and cancer. However, studies of RCAN1 in immunity are limited [131-132]. Second, it has to be noted that RCAN1 has a bidirectional role in regulating calcineurin activity depending on its expression level and phosphorylation status, which is not just a calcineurin inhibitor or stimulator [45]. Thus, precisely regulating RCAN1 activity needs to be carefully investigated. In addition, the isoform specific effect remains unclear. Moreover, the calcineurin-independent function is less studied. However, it is critical for developing RCAN1-based drugs.

## CONCLUSIONS

**Figure 1:**
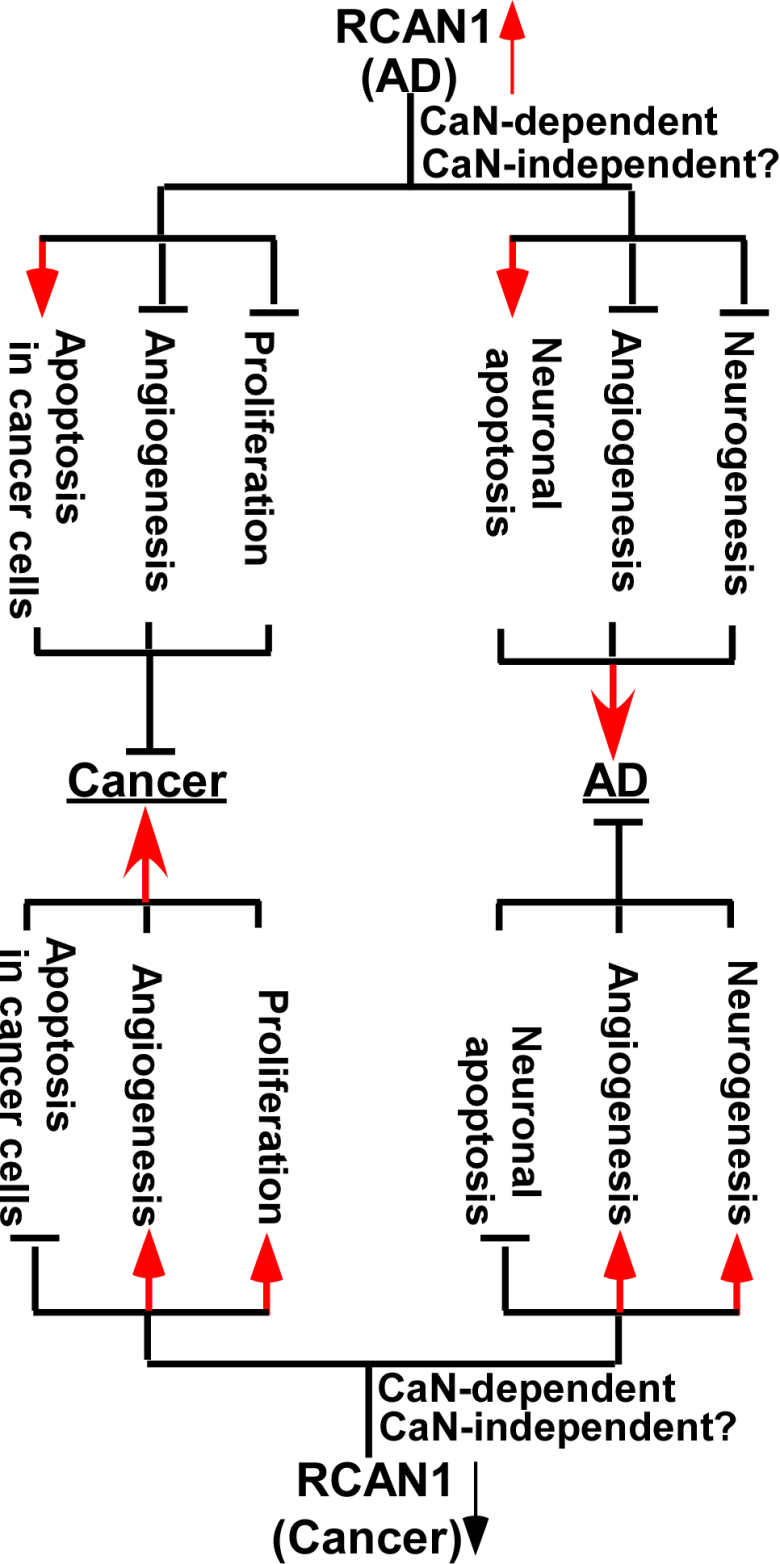
Mechanisms of RCAN1 in the inverse association between of Alzheimer’s disease and cancer Increased and decreased RCAN1 expression is detected in AD (top panel) and various types of cancer (bottom panel), respectively. Increased RCAN1 promotes AD pathogenesis by facilitating neuronal apoptosis, attenuating angiogenesis and inhibiting neurogenesis via calcineurin (CaN)-dependent or –independent pathways (top panel). However, increased RCAN1 inhibits cancer development by promoting cancer cell apoptosis, attenuating angiogenesis and inhibiting cancer cell proliferation via calcineurin-dependent or –independent pathways (top panel). On the other hand, reduced RCAN1 promotes cancer development by attenuating cancer cell apoptosis, facilitating angiogenesis and promoting cancer cell proliferation, while it inhibits AD pathogenesis by attenuating neuronal apoptosis, facilitating angiogenesis and promoting neurogenesis via calcineurin-dependent or –independent pathways (bottom panel).

The inverse association between Alzheimer’s disease (AD) and cancer has been reported in several population-based studies. Although the underlying mechanisms remain elusive, growing evidence indicates that RCAN1 is a key molecule of the inverse association. Increased and decreased RCAN1 expression are detected in AD and various types of cancer, respectively. Moreover, increased RCAN1 promotes AD pathogenesis by facilitating neuronal apoptosis, attenuating angiogenesis and inhibiting neurogenesis via calcineurin-dependent or –independent pathways. However, increased RCAN1 inhibits cancer development by promoting cancer cell apoptosis, attenuating angiogenesis and inhibiting cancer cell proliferation via calcineurin-dependent or –independent pathways (Fig. 1). On the other hand, reduced RCAN1 promotes cancer development by attenuating cancer cell apoptosis, facilitating angiogenesis and promoting cancer cell proliferation, while it inhibits AD pathogenesis by attenuating neuronal apoptosis, facilitating angiogenesis and promoting neurogenesis via calcineurin-dependent or –independent pathways (Fig. 1). Therefore, increased RCAN1 expression may contribute to the reduced incidence of some types of cancer in AD patients, while reduced RCAN1 expression may reduce the risk of AD in patients with various types of cancer. It suggested that dysregulation of RCAN1 plays a key role in the pathogenesis of both AD and cancer. Although precisely modulating the expression of RCAN1 may be a potential therapeutic target to treat AD and cancer, several key issues need to be resolved for drug development.
